# How Word/Non-Word Length Influence Reading Acquisition in a Transparent Language: Implications for Children’s Literacy and Development

**DOI:** 10.3390/children10010049

**Published:** 2022-12-26

**Authors:** Aparecido J. C. Soares, Fernanda C. Sassi, Talita Fortunato-Tavares, Claudia R. F. Andrade, Débora M. Befi-Lopes

**Affiliations:** 1 Department of Physical Therapy, Speech-Language Pathology and Audiology, and Occupational Therapy, Faculdade de Medicina, Universidade de São Paulo, Rua Cipotanea 51, São Paulo 05360-060, Brazil; 2 Department of Speech-Language-Hearing Sciences, Lehman College, City University of New York, New York, NY 10468, USA; 3 Ph.D. Program in Speech-Language-Hearing Sciences, The Graduate Center, City University of New York, New York, NY 10468, USA

**Keywords:** decoding skills, reading, literacy, reading development

## Abstract

Decoding skills are crucial for literacy development and they tend to be acquired early in transparent languages, such as Brazilian Portuguese. It is essential to better understand which variables may affect the decoding process. In this study, we investigated the processes of decoding as a function of age of children who are exposed to a transparent language. To this end, we examined the effects of grade, stimulus type and stimulus extension on the decoding accuracy of children between the ages of six and 10 years who are monolingual speakers of Brazilian Portuguese. The study included 250 children, enrolled from the first to the fifth grade. A list of words and pseudowords of variable length was created, based on Brazilian Portuguese structure. Children assessment was conducted using the computer program E-prime^®^ which was used to present the stimuli. The stimuli were programmed to appear on the screen in a random order and children were instructed to read them. The results indicate two important moments for decoding: the acquisition and the mastery of decoding skills. Additionally, the results highlight an important effect of the extent and type of stimuli and how it interacts with the school progress. Moreover, data indicate the multifactorial nature of decoding acquisition and the different interactions between variables that can influence this process. We discuss medium- and long-term implications of it, and possible individual and collective actions which can improve this process.

## 1. Introduction

In recent years, the study of reading development has been the focus of different areas given its relevance for academic success and its role as predictor of cognitive, intellectual, and linguistic achievement [1,2]. According to the theory of information processing [2,3], reading is a complex skill dependent of multiple linguistic-cognitive abilities, which, interdependently, act for the proper processing of decoded information [3,4]. Two routes, the phonological and the lexical, are responsible for the acquisition and development of reading [1,2,3,4]. Proficient reading is only reached when decoding is automatized and when cognitive and metacognitive mechanisms are available to enable the understanding of the decoded material [5,6,7]. The phonological route uses the grapheme-phoneme conversion process, translating letters or groups of letters into phonemes, through the application of grapheme-phonemic rules. In contrast, in the lexical route, pronunciation is not constructed segment by segment, but retrieved as a whole from the orthographic lexicon. However, the lexical route is used only when the item to be read has its orthographic representation pre-stored in the orthographic lexicon, that is, it was acquired through the phonological route [1,2,3,5,6,7]. Thus, development and automating of decoding process is fundamental for literacy [5,6,7].

In the process of acquisition and development of decoding and reading, there is a transition from slow reading, based on the grapheme-phoneme relationship, to rapid and assertive word recognition. This evidences the reduction in the use of the phonological route and the increase in the use of the lexical route [4,5,6,7]. Thus, phonological decoding is essential for the development of automatic visual recognition of words, a key skill for reading fluency. Initially, phonological decoding is responsible for familiarizing the novice reader with orthographic representations necessary for fluent and effortless decoding. It is important to emphasize, however, that this process occurs gradually, and the transition from the use of the phonological to the lexical route takes place throughout the development of reading and does not end with literacy [2,3,4,5]. Researchers from different areas have developed studies in order to verify the applicability of the dual route model to different languages, with conflicting results. The cognitivist model of reading, supported by the dual route theory, is also applicable to Brazilian Portuguese. Pinheiro [8] showed that beginning Brazilian readers tend to rely primarily on grapheme-phoneme conversion rules to decode unfamiliar words. Simultaneously, these readers acquire the orthographic representation of the decoded words so that they become familiar and, thus, the decoding can be automated.

In addition to the decoding process, another variable was identified to have great influence on the learning process of written code: the orthographic characteristics of each language [9,10,11,12]. Several studies have proved that such a process varies according to the orthography of the language in which the child is being literate [9,10,11,12]. Considering the orthographic variation of languages, a group of authors [13] developed the theory of orthographic depth. The authors argue that writing systems represent the phonology of a given language through orthography, via rules that do not necessarily occur on the phoneme-grapheme relation. Considering that writing systems represent the phonology of the language with different degrees of consistency, transparency (a phoneme is represented by a single grapheme and vice versa) or opacity (a phoneme is represented by more than one grapheme and vice versa) with which the relationship between phonemes and graphemes occurs in a language can facilitate or hinder the acquisition and development of reading. Following this line of research, later studies stated that, in alphabetic-based languages, such as Brazilian Portuguese, the development of reading begins with basic skills of grapheme-phoneme relation, followed by the acquisition of orthographic representations for a more automatic and fluent decoding [9,11,12,14]. Most studies on cross-linguistic differences in reading have focused on European orthographies [9,10,11,12]. Much less is known about other regions or even about languages that are variants from European languages, such as Brazilian Portuguese—which has its own characteristics as will be detailed in the following section. Thus, the findings on such languages would expand the knowledge on the reading acquisition process, including orthographies and variations of a language that share similarities with a European idiom, but which also has its exclusive characteristics.

### 1.1. Brazilian Portuguese Orthography

Although European Portuguese has an intermediate orthography depth (Seymour et al., 2003), Brazilian Portuguese has a very transparent decoding system, since it has only three inconsistent (irregular) graphemes [15]. The orthography of Brazilian Portuguese presents a set of consistent, biunivocal graph-phonemic relations and also the Brazilian pronunciation of vowels are very much different from European Portuguese since they are longer and more stressed, which facilitates its perception, consonants perception, phoneme-grapheme association and, thus, their decoding [15]. Moreover, the set of inconsistent graph-phonemic relations of Brazilian Portuguese are governed by rules, that is, they depend on the graphemic context, but are easily understandable by Brazilian Portuguese speakers [15]. Only a small part of this set of inconsistent relationships is not rule-governed, that is, they are irregular inconsistent. In this last set of inconsistent relations are found the three most opaque graphemes of Brazilian Portuguese. Thus, graphemes governed by rules independent of the graphemic context are: “p”, “b”, “t”, “d”, “f”, “v”, “ss”, “ç”, “sc”, “ch”, “j”, “nh”, “rr”, “ü”, “ó”, “õ”, “á”, “à”, “â”, and “ã”. Graphemes governed by context-dependent rules are described through 23 rules. For example, in this context, the rules for decoding the grapheme “g” in front of the letters that represent vowels, that is, “i”, “í”, “e”, “ê” and “é”, as in “gelo” and “girafa” (ice and giraffe) and also in other contexts, as in “água”, “gola” and “gato” (water, collar, cat).

There are decoding rules that depend on the application of metalinguistic knowledge or knowledge of the morphosyntactic and semantic context present in the text. However, in some of these cases, knowledge must be combined with the pairing of the word with the orthographic representation present in the mental lexicon. These items, therefore, can only be read correctly via lexical route. In this last set are the rules for decoding the graphemes “e” and “o” when not marked by a diacritic. The same applies the grapheme “x” in intervocalic position, as it can represent three different sounds: /S/ as in “abacaxi” (pineapple), /s/ as in “máximo” (maximum) and /ks/ as in “taxi”. The correct decoding of this grapheme, in these contexts, depends on the storage of orthographic representations of the words in the mental lexicon.

### 1.2. Decoding Assessment

Decoding is a crucial skill for literacy and for the consolidation of fluent reading and, consequently, reading comprehension [16,17,18]. The assessment of decoding through read aloud is currently the most frequently used measure to monitor the acquisition and progress of the skill, both with regard to school assessments and to verify the effectiveness of intervention programs [19,20,21]. In addition, the results of the oral decoding assessment are an important predictor of the reading performance of the individual. In the United States, oral reading assessment measures are analyzed by the Federal Education Department to monitor the academic development and to develop stimulation and/or intervention programs [19]. The type of material used for the evaluation must be adequate to the objective that has been set, as the results differ according to measures, such as isolated words or texts [19,20,21]. The oral reading of isolated words is the most frequently used task to assess the individual’s proficiency in decoding [22]. This task isolates context or visual (pictorial) cues and thus strictly evaluates decoding. The reading assessment models are strongly based on the dual route model, with the use of words and nonwords.

In Brazil, it is common for schoolchildren to present some difficulty in reading or writing [23]. Therefore, it is essential to characterize the reading condition of children to allow proper identification of typical variations of development or possible deficits. According to the latest evaluation of the Program for International Student Assessment (PISA), Brazil remained with high rates of school failure [24] and the country has been among the worst performing countries for 10 years. According to this latest report, the reading difficulties faced by Brazilian schoolchildren begin in elementary education, interfering with the consolidation of literacy. These difficulties, when not identified or treated, become chronic, leading the student to low performance throughout the school years.

In view of this reality, the occurrence of “false positives” for reading and learning disorders is very common, since the characteristics of a learning difficulty can resemble the manifestations observed in different learning disorders and specialized professionals need to carry out the appropriate differentiation of these conditions, in a specific evaluation and with the support of a multidisciplinary team. Thus, decoding assessment becomes essential, as it allows early identification of possible deviations in development, elaboration of stimulation and rehabilitation programs, in addition to favoring the adequate process of literacy and schooling. Furthermore, understanding how language characteristics (i.e., opacity/transparency) facilitate or hinder such a process is of great value to different areas of knowledge and countries so that public policies to promote literacy can be specifically strengthened and advanced [10,11].

### 1.3. Empirical Implications

In addition to understanding the development of decoding of children in literacy process ages in terms of opacity and transparency, advancing investigations by understanding how the characteristics of a language itself (i.e., word length/syllable structure) are of fundamental importance to expand understanding in the area [5,6].

In recent years, studies have investigated the reading processing in relation to other aspects of the language, such as word length and syllabic structure [10,11,12]. The syllabic structure of French interferes both in the development of decoding and in spelling knowledge on the graph-phonemic decoding and writing of children [12]. For Brazilian Portuguese [21], children with less schooling have trouble in decoding words that are longer or outside the most common standard of the Portuguese language (Consonant-Vowel). It is important to highlight that the study [21] was limited to two grades and the authors claimed that more studies in the field were needed. Such data may bring consistent subsidies for the planning and execution of actions that can, in the medium and long term, reduce the low reading rates commonly presented by Brazilian students. 

Understanding the acquisition patterns of graph-phonemic decoding and reading in the different spelling patterns is important, not only to favor the development of children according to their language, but also to identify in which way the predictors of the reading skills vary from one language to another [9,10,11]. Continuity and research advances investigating linguistic features more deeply can also promote a better understanding of how these features correlate with the underlying decoding skills (i.e., phonological awareness, RAN) to them in different languages [9,10,11,12].

### 1.4. The Present Study

The present study is anchored in the theory of information processing and considers the theoretical assumptions that support the theory of double route as well as its interaction with the different characteristics of the languages. The aims of this study are to investigate the effect of grade, stimulus type (word/nonword) and stimulus extension on the decoding accuracy of children between six and 10 years of age who are speakers of Brazilian Portuguese through a list of words and pseudowords that considers the characteristics of the language. The present study is fundamental for the deepening of knowledge on the process of acquiring basic reading skills and its relationship with the characteristics of a transparent language. In addition, the current study will provide data for transversal and longitudinal cross-linguistic studies in different fields. It is noteworthy that this study differs positively from others by studying the effects of different linguistic characteristics on the literacy process of children in an entire literacy cycle, in addition to providing data on two of the most used reading assessment measures [16,17,18,19].

### 1.5. Hypotheses

**Hypothesis** **a.** *As the decoding acquisition process develops, better performance is expected for older children, shorter stimuli, and words, as compared to nonwords. Grade (a1), stimulus type (word/pseudoword) (a2), and stimulus length (a3) will influence performance on the reading task.*

**Hypothesis** **b.** *The following interactions are expected:*

**Hypothesis** **b1.** *Between grade and type of stimulus: that is, the differences in decoding performance between words and pseudowords will vary according to grade. Based on the dual route hypothesis, we expect the difference to be greater in lower grades than in higher grades.*

**Hypothesis** **b2.** *Between grade and stimulus length: that is, the difference in reading performance between monosyllables and polysyllables will vary according to grade. As decoding acquisition becomes more advanced, the effect of stimulus length should be less pronounced and, therefore, expected to be smaller for higher grades.*

**Hypothesis** **b3.** *Between stimulus type and length: that is, the difference in reading performance between monosyllables and polysyllables will be different for words and pseudowords. Based on the dual route hypothesis, longer nonwords should be more challenging than longer words as no benefit from lexical route is expected for nonwords.*

**Hypothesis** **b4.** *Between grade, stimulus type and stimulus length: that is, the interaction between grade and word length should be different for each type of stimulus as the decoding acquisition process advances.*

## 2. Material and Methods

This is a prospective study that followed the principles of the Standards for Educational and Psychological Testing (SEPT) [25], a guideline proposed by American organizations that compiles fundamental recommendations and definitions regarding the psychometric aspects involved on the preparation and interpretation of tests, in addition to the different necessary steps for validation of a procedure. This study was approved by the Institution’s Research Ethics Committee (CEP No. 2262300). The data collection procedures started only after schools, parents/guardians, and children signed the Free and Informed Consent Form.

### 2.1. Procedures

#### 2.1.1. Step 1: Evidence of Validity Based on Test Content

At this stage, the target population was defined, an extensive literature review was carried out, and, for the elaboration of the items, the syntactic and semantic aspects that contribute to the clarity, pertinence, coherence and scope of the items were considered. The representativeness and relevance of the items in relation to the outcome was evaluated by judges with expertise in the subject of the test.

Definition of the target population: students from a public and a private school, both in the city of São Paulo, were included in the study with the aim of evaluating a representative sample of school-age children. The indicators of the National Institute of Studies and Research (INEP) in relation to the test *Provinha Brasil*—which is the main indicator for calculating the Basic Education Development Index (IDEB)—were considered to select the schools to be included in this study. The selected schools presented scores close to that observed in the national average for public and private schools based on the most recent published data [23].

Literature Review: an extensive literature review was carried out regarding the different word reading tests or word banks developed for Brazilian Portuguese in recent years [26,27,28,29,30,31,32,33]. It was observed that most of the compiled literature used criteria such as frequency of words, or even their concreteness, with the exception of one study [33] that was based on the language decoding rules [15]. We emphasize, however, that we did not find tests or procedures designed according to the characteristics of Brazilian Portuguese that considered aspects beyond the decoding rules, such as: variation in word length and its frequency of occurrence in the language.

Elaboration of the items: a list of words and nonwords of variable lengths (from monosyllabic to polysyllabic) was created, based on three fundamental principles: (a) the decoding rules of Brazilian Portuguese [15,34]—context-independent graphophonemic correspondence, context-dependent graphophonemic correspondence, and irregular graphemes; (b) length variability of Brazilian Portuguese words—words ranging from monosyllables to polysyllables; (c) the frequency of occurrence of the different word length in the language [34], as shown in Table 1.

In Brazilian Portuguese, 86.1% of words are concentrated between mono and polysyllables with a maximum of five syllables. For this reason, words of the present study followed this same pattern. Elementary school children, the target population of this procedure, are not exposed to all variations in Brazilian Portuguese word length [15,20]. Therefore, polysyllables with up to five syllables were included. Taking into account the decoding rules, the length of words, and the frequency of occurrence of these words in the children’s experiences, a final list with a total of 68 words distributed as follows was created: 6 monosyllables (8.8%) (i.e., *boi (ox), pé (foot)*), 16 two-syllables (23.5%) (i.e., *noite* (night)*, chuva* (rain)), 22 tri-syllables (32.3%) (i.e., *escola* (school), *zeloso* (zealous)); 16 polysyllables with up to four syllables (23.5%) (i.e., *aquarela* (watercolor), *nascimento* (birth)), and eight polysyllables with five syllables (11.7%) (i.e., *maravilhosa* (wonderful), *insegurança* (insecurity)*)*. (Appendix A).

The use of nonwords in the decoding assessment of Brazilian Portuguese speakers is essential due to the transparency of the language, especially when considering the dual route [15,34]. The list of nonwords was designed by a linguist based on the list of words, respecting the phonological structure of each one of them. The following rules were adopted:

- Vowels—(a) always keep the corresponding low for exchange (/a/in an unstressed position); (b) replace the middle vowel with a middle vowel; (c) replace the high vowel with a high vowel;
- Plosives/Fricatives—replace respecting the following order of priority: point of articulation, voicing and, in case of impossibility, mode of articulation;
- Nasal—replace only the point of articulation;
- Liquid—replace lateral phonemes with non-lateral ones and vice versa.

In addition, the transformation of words into nonwords followed criteria for maintaining the length of the word. Thus, for the monosyllables, only the vowels were changed; for two-syllables, a vowel and a consonant were changed; for trisyllables, two consonants and one vowel were changed; for polysyllables, three consonants and two vowels were changed. After changes were made to the structure of the words, they were spelled in order to respect the decoding rules of Brazilian Portuguese. However, the various possibilities of graphophonemic representation were considered, since the nonwords do not follow the orthographic rules of the language (Appendix B).

Analysis by expert judges: each of the nonwords were evaluated by three different expert judges who determined whether the nonwords were adequate to the instrument’s construction criteria, both in terms of structure and length. The collected data were submitted to statistical analysis using the SPSS software version 25. The analysis of agreement between judges was performed based on the value of Fleiss’ Kappa coefficient, which is a generalization of Cohen’s Kappa coefficient. Kappa coefficient values greater than 0.75 are considered to indicate excellent inter-judge agreement; between 0.40 and 0.75 as moderate; and below 0.40 as weak and/or non-existent. In this research, the analysis of agreement among the three judges showed the following results: k = 0.800 for adequacy to the nonword structure criteria (excellent agreement) and k = 0.575 for adequacy to the non-word extension criteria (moderate agreement).

The developed list of words and nonwords will henceforward be addressed as The Protocol for Decoding Acquisition Development—*Protocolo de Acompanhamento do Desenvolvimento da Decodificação* (PRADE) [35] (Appendix A and Appendix B).

#### 2.1.2. Step 2: Evidence of Validity Based on Response Processes

In this step, the adequacy, structure and application of the items in a real context were verified. There is no explicit recommendation for the sample size at this stage. It is suggested the formation of representative strata of the target population, composed of at least 10 individuals in each stratum. Interviews/procedures were carried out to verify that participants understood the test items [25].

*Sample size*: the number of classes (strata or groups) into which the sample would be divided was considered. Thus, the formula below was adopted to calculate the minimum sample size (considering k = 1 + 3.322 × logn where; k = number of classes (strata or groups); n = sample size; log = base 10 logarithm):

(1) $$\mathrm{logn}=\frac{k-1}{3.322}\;\Rightarrow n=10^{\frac{k-1}{3.322}}$$

Considering that each school would have five distinct groups of children (grades 1 to 5), the minimum number of sample elements determined for each group was 16. Thus, it was established that the sample should have at least 80 sample elements, distributed among the school groups.

To guarantee the statistical power of the sample we chose to collect a number greater than the minimum indicated by the analysis. In addition, we selected a balanced number of children from public and private schools previously selected in order to constitute a representative sample of the Brazilian educational reality. Thus, the study included 250 children, enrolled from first to fifth grade of elementary school. Each grade had 50 children, as follows: 23 girls and 27 boys in 1st grade, with a mean age of 6.6; 23 girls and 27 boys in 2nd grade, with a mean age of 7.7; 22 girls and 28 boys in 3rd grade, with a mean age of 8.5; 26 girls and 24 boys in 4th grade, with a mean age of 9.6; and 21 girls and 29 boys in 5th grade, with a mean age of 10.5.

To ensure that the study sample was composed of children with different academic profiles and to prevent a single profile of children from being indicated for participation, we chose to use stratified random sampling for the selection of participants. Thus, the children were numbered from 1 to 250, in ascending order, according to the school year, and then these numbers were used to randomly select the final sample of the study.

Participants are able to complete the procedures: to be included in the study, children should have no auditory or visual complaints; no signs of neurological or cognitive disorders; absence of retention in school records; no phonological and oral language alterations. Oral language was assessed through the ABFW phonology test [36], which consists of naming and imitating phonologically balanced linguistic items. In addition, we also applied the word reading subtest of the School Performance Test [37] due to its procedural similarity with what was intended to be evaluated in this research.

#### 2.1.3. Step 3: Evidence of Validity Based on Internal Consistency

In this step, the degree of relationship between the test items and the outcome was verified by applying the test to a sample of the target population. Corrected item-total correlation and inter-item correlation was observed [25].

Application of the test in a sample of the target population: for this stage, the computer program E-prime^®^ was used to present the stimuli. The stimuli were programmed to appear on the screen in random order. Before starting the experiment, each of the 250 children was presented with a screen containing instructions about the test, which were read by the researcher: “Hello! Next, words that exist and that do not exist, of different sizes, will appear. Read them aloud the way you think the word should be read. If a word that you do not know appears, no problem, move on to the next one! Good reading”—*Olá, agora eu vou te apresentar palavras que existem e que não existem, de diferentes tamanhos. Leia em voz alta do jeito que você acha que a palavra deve ser lida. Se aparecer alguma palavra que você não conhece, você pode pular para a próxima, sem problemas. Boa leitura!-* The stimuli to be decoded were typed in Arial font, size 20, in uppercase. The children were instructed to read the words the way they were used to or the way they thought they should be read. If the child refused to read the word or could not decode it, they could skip it. The experiment was designed and run on E-Prime and video recorded for posterior analysis. Reading time was computed by E-Prime. Transcriptions of the responses were conducted by two Speech-Language Pathologists and the score of 0 was assigned to incorrect decoding and the score of 1 was assigned for correct decoding. No discrepancies were observed. As expected, the analysis of data from older children was faster and easier to compute than children from first and second grade given their more advanced decoding skills.

Data analysis: to verify the decoding accuracy, only the percentage of words correctly read was considered, respecting the graphophonemic and orthographic relations, in the case of words, and the graphophonemic relations, in the case of nonwords. Such data were also analyzed both in terms of the length of the words and the total percentage of correct answers in each of the lists. Generalized Estimated Equations (GEE), a method for modeling clustered data, was applied to estimate the parameters of a generalized linear model with a possible unmeasured correlation between observations and test the study hypotheses.

## 3. Results

**Figure 1:**
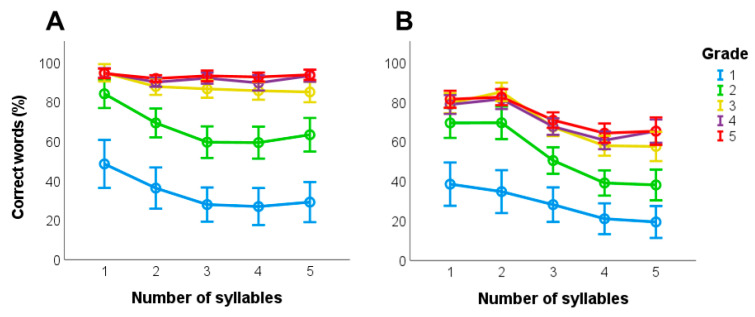
Percentage accuracy according to grade and number of syllables of words (**A**) and nonwords (**B**). Error bars represent 95% confidence interval.

Figure 1 shows the mean percentage of correct responses (and 95% confidence intervals) according to grade and the number of syllables for words and nonwords. In general, the percentage of correct responses was higher for more advanced grades, with first and second grades differing between them and among the others. The percentage of correct responses decreased with increasing stimulus length, but more markedly when comparing monosyllables, disyllables and trisyllables, especially in the early grades, as expected. A better performance for words compared to nonwords was observed.

Complete measures of central tendency and dispersion on the percentage of correct responses according to grade, stimuli type and length can be found in Table 2. Data show, in general, better performance in reading as the scholar grade advances. It should be noted that for first and second grade, the effect of word length and stimuli were greater than that observed in the more advanced grades, as expected. In addition, the standard deviation tends to decrease as the school grade advances indicating more homogeneity from the third grand onwards. The results from the pseudowords demonstrate the same pattern of decoding skills, although with a decrease in the accuracy percentage in all grades when compared to the words, except from the first grade.

A Generalized Estimated Equations (GEE) model was applied to test the study hypotheses and verify the effect of grade, type and length of stimulus (Hypothesis a) and two-way and three-way interactions between these variables (Hypothesis b) on the percentage of correct answers in the reading task. Based on the nature of the dependent variables, the best fit was obtained considering a gamma distribution with identity link function and an unstructured covariance matrix for the two variables testing different adjustments based on the quasi-likelihood under the Independence Model criterion (QIC) and evaluating the model residuals using Q-Q graphs. Table 3 presents the effects of each factor separately for each of the models.

Table 4 presents the complete model with parameter estimates for each main effect. The effect size was measured by calculating the d coefficient based on the proposal by Feingold (2009).

Taken together, the results of Table 3 and Table 4 demonstrate that there are multiple effects and interactions between the variables that these factors influence the accuracy on a reading task.

To better investigate the observed effects, post-hoc analyses of the estimated marginal means of percentage of correct responses for each grade and stimulus length and type was conducted using Student’s *t*-tests with Bonferroni correction for multiple comparisons. The effect size was measured by calculating the d coefficient (Cohen, 1992). The results of these tests can be found in Table 5.

The data in Table 5 indicate a high variability in responses from children in lower grade (1st grade) and the effect of word length is limited to the 2nd grade, which is quite different from all other grades. The data also indicate a ceiling effect of stimuli length on decoding performance of words around third grade, contrary to what is observed for nonwords, which maintains such an effect until fifth grade.

## 4. Summary of Findings

### 4.1. Hypothesis A

We observed that (a1) school year (X^2^ = 157.101, df = 4, *p* < 0.001), (a2) type of stimulus (word/pseudoword) (X^2^ = 727.674; df = 1; *p* < 0.001), and (a3) word length (X^2^ = 485.817; gl = 4; *p* < 0.001) influenced performance in the decoding task, confirming hypothesis A. Better performance was observed the higher the grade, the shorter stimuli, and with the presentation of words as a type of stimulus as the decoding acquisition process advanced.

### 4.2. Hypothesis B

All hypotheses related to interactions were confirmed, indicating that there are significant interactions between (b1) grade and stimulus type (X^2^ = 126.102, gl = 4, *p* < 0.001) that is, the difference in decoding performance between words and nonwords is greater as grades advances, supporting the dual route theory; (b2) grade and stimulus length (X^2^ = 101.155, gl = 16, *p* < 0.001) that is, the difference in decoding performance between monosyllables and polysyllables should decrease as grades advances, as evidence of the advance in the decoding acquisition process; (b3) stimulus type and length (X^2^ = 379.190, gl = 4, *p* < 0.001) that is, the difference in decoding performance between monosyllables and polysyllables is different for words and nonwords, also supporting the dual route theory, as longer nonwords should be more challenging than longer words as no benefit from the lexical route is expected for nonwords; (b4) grade, type of stimulus and length of stimulus (X^2^ = 115.962, df = 16, *p* < 0.001) that is, the interaction between grade and word length is different for each type of stimulus as the decoding acquisition process advances.

## 5. Discussion

The current study investigated the processes of decoding as a function of the age of children who are exposed to a transparent language. The effects of grade, stimulus type and stimulus extension on the decoding accuracy of children between the ages of six and 10 years who are monolingual speakers of Brazilian Portuguese were studied. The findings from this study are in line with different studies that stated that in alphabetic-based languages, such as Brazilian Portuguese, the development of decoding tends to be early [9,10,11,12,13,14,38,39]. Furthermore, the multiple interactions between the variables investigated in this study are in line with international research that indicates the multifactorial nature of decoding development [6,40,41,42]. This fact is extremely important for the understanding of the acquisition process so that investment in projects and public policies that better direct the literacy process can be fulfilled.

### 5.1. Acquisition of Decoding Skills

The results indicate two important moments for decoding development: the acquisition phase, in first and second grade; and the mastery phase, in third grade, with similar performance in fourth and fifth grades. In addition, there is an important effect of length and type of stimuli and how they interact as grade progresses, with such effect reducing as grade advances. Regarding nonwords, there is a greater influence of length, with a lower percentage of accuracy throughout the entire elementary school cycle when compared to words.

The pattern of decoding development observed in this study is in accordance with that described by the dual route theory [1,2,6], which explains the development of automaticity in reading. According to that theory, as individuals learn to decode and master the written code, they tend to show major changes in their decoding characteristics at the beginning of the process. Thus, as their orthographic lexicon increases, the values found in the decoding speed tend to stabilize and the differences with their peers of similar grades are reduced [10,11].

Bar-Kochva and Breznitz [43] also argue that for children learning to decode in more transparent orthographies, understanding and mastering the grapheme-phoneme conversion rules, in addition to providing faster learning of written code, will initially imply a greater dependence on phonological skills than on those of visual recognition. In the present study, the phonological route was strongly influenced by word length, as there was a decline in the percentage of correct responses with increasing stimuli length, especially with regard to nonwords. In the case of words, this influence strongly concentrated in first and second grades, attenuating from the third grade onwards. This data is extremely important because it allows reflections on literacy methods, educational speech therapy programs, and even the therapeutic intervention of children with learning disabilities, indicating that the length of stimuli should be an important variable part of the planning of activities for children in the initial phase of the decoding acquisition process.

Caravolas [10] found similar results in a study carried out with English, Czech and Slovak speaking children when identifying greater gains in speed and accuracy in words when compared to nonwords. The authors argue that that finding may be due to the fact that nonwords are of low frequency, while words tend to be stored in the orthographic lexicon of readers, providing direct access and, consequently, faster decoding. The author, however, reaffirms the importance of the phonological route for the acquisition of orthographic patterns in alphabetic-based languages, reinforcing the hypothesis of “self-teaching” provided by learning the graph-phonemic conversion rules.

In line with what was observed in this study, more recent research has shown that, in general, decoding measures (i.e., accuracy and speed) tend to be more heterogeneous between the first and third grades of elementary schooling, as opposed to what happens between the third and fifth grades, which tend to be more stabilized and even similar [44]. It is noteworthy that this pattern of development finds theoretical support from the dual route theory, which explains the development of automaticity in reading [2,5,6,7]. Thus, as individuals learn to decode and master the written code, they tend to present greater changes in reading characteristics at the beginning of the process. As their spelling lexicon increases, the values found in the decoding speed tend to stabilize and the differences among their peers of close grades are reduced [44,45,46]. Thus, the importance of encouraging the development of grapho-phonemic conversion skills in the initial grades is reinforced so that the process of acquisition and development of decoding and reading are enhanced both in clinical and institutional settings. At the same time, for children from third grade onwards, the data suggest the need to reinforce activities beyond decoding, which also involve aspects related to reading fluency and its prosodic aspects and comprehension in order to concretize and improve the development of this very important skill, fundamental for the development of the individuals in all spheres of their lives.

### 5.2. Decoding Skills, Policies e Social Economical Status

The present study indicates that for children literate in Brazilian Portuguese, the acquisition of decoding occurs primarily between the first and third grades, with increasing automatization and mastery of the lexical route onwards. These data indicate the third grade as an important highlighter in reading development of Brazilian schoolchildren and are in agreement with international studies that point out that, in transparent languages, the development of decoding tends to happen early and stabilize in more advanced grades [12,13,14]. The fundamental importance of mastering decoding for the development of reading as a whole is well known [12,13,14,39,43], thus, these data can be an important indicator for the development of public education policies aiming to improve teaching methods.

The document from the Brazilian Ministry of Education that determines the National Curriculum Bases indicates that around third grade students must master decoding [47]. However, the latest reports from the Program for International Student Assessment PISA [24] indicate that the performance of Brazilian children evaluated in reading comprehension is flawed throughout the education chain, mainly due to the residual difficulties in decoding that these children carry in their trajectory. Ultimately, these residual difficulties make reading comprehension difficult and compromise the children’s general academic performance.

This effect may be exacerbated by other factors such as Social Economic Status (SES). Kainz [48] discussed the academic outreach of African-American and Latin American children from schools in low-socio-economic neighborhoods in the United States. The study included twenty thousand children enrolled in 900 schools across the United States as a sample and carried out an evaluation of these children at the end of kindergarten and at the end of the first grade. The data showed evidence that programs from the United States Department of Education aimed at reducing the academic differences of these children with their peers in privileged situations proved to be highly effective in reducing deficits and gaps found on the first assessment of these individuals. The author also stated that the smaller number of children per classroom and teachers with better training were decisive variables for the data found. The strengthening of public education policies, teacher training, and a broader presence of educational speech therapy can be crucial elements for the increasing the potential of public education in Brazil. Considering the current findings regarding the role of word length on the acquisition of decoding in Brazilian Portuguese, the development of didactic materials, teacher training programs, and strategies to reduce learning difficulties must consider this variable to guarantee greater success in the initial grades and thus, promote the development of better readers with a positive impact in the Brazilian educational reality.

### 5.3. Cross-Linguistic Comparisons

Our data points that in transparent languages, such as Brazilian Portuguese, reading accuracy is influenced by word length, especially at the beginning of the schooling process. There are similar results described for other transparent languages such as Finnish, Greek, German, Czech and Italian [10,11,12,39,42,43,49], in which the variations of this early domain of decoding according to the degree of transparency are discussed. In some languages, such as Finnish, it is possible for children to master decoding in their first year of school [50]. In the case of Brazilian Portuguese, a language considered transparent, such mastery only occurs in the third year, although it is possible to verify a better performance of the second year compared to the first. Such data is of fundamental importance to better understand the process of acquiring decoding in the different ranges of transparency of the languages in which this process is studied. Bar-Kochva and Breznitz [43] also suggest that in learning to decode languages with transparent orthography, understanding and mastering the grapheme-phoneme conversion rules favor the learning of the written code, with a greater dependence on phonological skills than on visual recognition. In transparent languages, phonological skills not only favor the learning of the written code, but also help improve reading with a more assertive decoding [10,11,12].

We can hypothesize that in transparent orthographies, monitoring the acquisition of decoding in typical children should be a priority in the initial grades, with the aim of automating this process at the beginning of literacy. Decoding is essential for the development of reading fluency and, consequently, reading comprehension, which is the final objective on the domain of written code [10,12,18,46]. Thus, monitoring the acquisition of decoding becomes essential, especially in developing countries where educational indexes are generally low on international assessments. In the latest PISA report [24] for example, the data indicated that Brazil has remained stagnant in the past decade, and Brazilian readers have consistent reading deficits. The report details that most students fail to learn basic elements of reading, such as decoding [24].

The present study recruited subjects only in the city of São Paulo, which is the most populous city in Brazil, with the population of about 22 million in the metro area. Further studies in other regions of the country are needed, mainly considering the extension of Brazil and the variation in social and educational opportunities according to different regions. Considering the reading route theory in which this study is anchored, we believe that the pattern of decoding acquisition may be very similar for different regions. However, children from underprivileged regions may acquire and master decoding skills later than observed here. That is a fundamental question that must be addressed in further studies.

## 6. Conclusions

The current study contributes to the advancement of understanding of the decoding acquisition process by children who speak Brazilian Portuguese, and are literate in transparent orthographies languages, by showing that the acquisition of decoding is influenced by the type and length of the stimulus and that this influence varies according to the elementary school grades, evidencing the dual route theory [1,2,3,4,5], which has both clinical and theoretical implications.

The current findings contribute significantly to the area by indicating not only the process of acquiring decoding in a transparent language, but also its multifactorial nature and the different interactions between variables that can positively or negatively influence this process. These data are fundamental for the expansion of this process in a transparent language, but we also discuss their medium- and long-term implications, including possible individual and collective actions for the improvement of this process, mainly when considering the importance of decoding for literacy and for further development of the individual.

## Figures and Tables

**Table 1:** Distribution of Brazilian Portuguese words according to number of syllables, according to REF 35.

Number of Syllables	Number of Words	Percentage
1	546	0.3
2	11,712	7.7
3	36,790	24.3
4	48,218	31.9
5	33,125	21.9
6	13,926	9.2
7	4665	3.09
8	1440	0.95
9	362	0.23
10	76	0.05
>10	16	0.01

**Table 2:** Descriptive values of percentage accuracy according to grade and stimuli type and length.

Grade	Stimuli Length	Stimuli Type	N	Mean	SD	CI 95%		Median	Minimum	Maximum
						UL	LL			
1st Grade	Monossyllable	Word	50	48.67	42.97	38.00	59.29	33.33	0.00	100
		Nonword	50	38.67	38.60	29.33	48.00	33.33	0.00	100
	Dissyllable	Word	50	36.38	36.89	26.75	45.80	12.50	0.00	93.75
		Nonword	50	34.88	38.28	25.13	44.13	12.50	0.00	93.75
	Trissyllable	Word	50	28.00	30.60	20.00	36.36	11.36	0.00	86.36
		Nonword	50	28.27	30.79	20.50	35.41	11.36	0.00	86.36
	Polissyllable (4)	Word	50	27.00	33.15	18.29	35.33	0.00	0.00	93.75
		Nonword	50	21.12	27.46	13.75	28.75	0.00	0.00	87.50
	Polissyllable (5)	Word	50	29.25	35.86	20.75	37.68	0.00	0.00	100
		Nonword	50	19.50	28.48	12.50	27.45	0.00	0.00	100
	Total	Word	50	30.77	32.63	22.17	39.15	10.00	0.00	90.00
		Nonword	50	27.23	30.03	19.34	34.62	8.57	0.00	81.43
2nd Grade	Monossyllable	Word	50	84.33	25.51	76.67	91.33	100	0.00	100
		Nonword	50	69.67	26.66	62.00	76.67	75.00	0.00	100
	Dissyllable	Word	50	69.50	25.77	62.25	76.19	81.25	0.00	100
		Nonword	50	69.75	29.08	61.88	77.38	78.13	0.00	100
	Trissyllable	Word	50	59.73	28.26	52.35	66.28	65.91	0.00	100
		Nonword	50	50.64	23.61	43.82	57.36	52.27	0.00	86.36
	Polissyllable (4)	Word	50	59.50	28.63	51.42	67.41	62.50	0.00	100
		Nonword	50	39.25	22.52	33.13	45.75	37.50	0.00	87.50
	Polissyllable (5)	Word	50	63.50	30.08	54.50	72.00	68.75	0.00	100
		Nonword	50	38.25	27.36	31.50	46.00	37.50	0.00	100
	Total	Word	50	62.74	25.50	55.24	69.22	72.14	0.00	92.86
		Nonword	50	51.17	22.97	44.72	57.63	53.57	0.00	88.57
3rd Grade	Monossyllable	Word	50	95.00	15.52	90.00	98.33	100	0.00	100
		Nonword	50	79.67	17.91	74.67	84.00	83.33	16.67	100
	Dissyllable	Word	50	88.00	14.88	82.88	91.50	87.50	0.00	100
		Nonword	50	85.38	16.49	80.13	89.25	87.50	0.00	100
	Trissyllable	Word	50	86.82	15.81	81.73	90.55	90.91	0.00	100
		Nonword	50	67.55	15.99	62.73	71.81	68.18	0.00	90.91
	Polissyllable (4)	Word	50	85.88	16.01	80.50	89.88	87.50	0.00	100
		Nonword	50	58.13	17.73	52.75	63.00	62.50	0.00	87.50
	Polissyllable (5)	Word	50	85.25	18.33	79.75	90.00	87.50	0.00	100
		Nonword	50	57.75	26.11	50.25	65.25	62.50	0.00	100
	Total	Word	50	84.91	14.13	79.83	88.27	87.86	0.00	95.71
		Nonword	50	67.43	14.89	62.75	71.51	70.00	0.00	85.71
4th Grade	Monossyllable	Word	50	95.00	8.42	93.00	97.00	100	66.67	100
		Nonword	50	79.00	16.77	74.67	83.00	83.33	33.33	100
	Dissyllable	Word	50	90.25	8.30	87.86	92.63	93.75	62.50	100
		Nonword	50	81.75	17.76	76.75	86.63	87.50	31.25	100
	Trissyllable	Word	50	92.27	9.94	89.36	95.00	95.45	59.09	100
		Nonword	50	67.82	14.75	63.23	71.95	68.18	27.27	100
	Polissyllable (4)	Word	50	89.88	13.65	86.00	93.50	93.75	37.50	100
		Nonword	50	60.87	15.86	56.77	65.25	62.50	12.50	100
	Polissyllable (5)	Word	50	93.50	10.79	90.50	96.25	100	50.00	100
		Nonword	50	65.50	20.76	60.25	70.75	68.75	12.50	100
	Total	Word	50	89.00	7.96	86.54	91.19	91.43	60.00	97.14
		Nonword	50	68.17	13.23	64.21	71.93	71.43	22.86	91.43
5th Grade	Monossyllable	Word	50	94.67	8.54	92.33	96.67	100	66.67	100
		Nonword	50	81.67	15.15	78.00	85.33	83.33	50.00	100
	Dissyllable	Word	50	92.13	5.90	90.50	93.50	93.75	75.00	100
		Nonword	50	82.75	14.32	78.75	86.75	87.50	43.75	100
	Trissyllable	Word	50	93.45	9.55	90.45	96.09	95.45	54.55	100
		Nonword	50	71.09	13.80	67.64	74.55	72.73	22.73	100
	Polissyllable (4)	Word	50	92.88	7.99	90.28	95.00	93.75	62.50	100
		Nonword	50	64.50	17.19	59.38	69.37	68.75	18.75	87.50
	Polissyllable (5)	Word	50	94.00	9.19	91.50	96.25	100	62.50	100
		Nonword	50	65.50	24.43	59.25	71.75	62.50	12.50	100
	Total	Word	50	90.51	5.73	88.77	92.14	92.86	72.86	97.14
		Nonword	50	70.49	11.29	67.45	73.83	72.14	45.71	92.86

Note: SD = standard deviation; CI = 95% confidence interval calculated with 1000 *bootstrap samples*; LL = lower limit; UL = upper limit.

**Table 3:** Effects test for each GEE factors for accuracy and percentage of correct responses according to the hypotheses of the study.

		Effects						
	Intercept	Hypothesis A1 Grade (GR)	Hypothesis A2 Stimuli Type (ST)	Hypothesis A3 Stimuli Length (SL)	Hypothesis B1 GR × ST	Hypothesis B2 GR × SL	Hypothesis B3 ST × SL	Hypothesis B4 GR × ST × SL
X^2^ Wald	2,924,078	157,101	727,674	485,817	126,102	101,155	379,190	115,962
DF	1	4	1	4	4	16	4	16
*p*	<0.001 *	<0.001 *	<0.001 *	<0.001 *	<0.001 *	<0.001 *	<0.001 *	<0.001 *

Test X^2^ Wald. Note: DF = degrees of freedom; * = statistically significant value 5% (*p* ≤ 0.05).

**Table 4:** Estimated GEE effects of grade, stimuli type and length on percent accuracy.

		Comparison	b	Standard Error	95% CI		X^2^ Wald	*p*	Effect Size
					LL	UL			
Percent accuracy	Intercept	-	49.67	6.02	37.88	61.46	68.168	<0.001 *	1.538
	Grade	1° vs. 5°	46.00	6.13	33.98	58.02	56.250	<0.001 *	1.425
		1° vs. 4°	46.33	6.13	34.32	58.35	57.132	<0.001 *	1.435
		1° vs. 3°	46.33	6.40	33.80	58.87	52.477	<0.001 *	1.435
		1° vs. 2°	35.67	7.00	21.95	49.38	25.991	<0.001 *	1.105
	Stimuli type	Word vs. Nonword	−10.00	2.49	−14.89	−5.11	16.071	<0.001 *	0.310
	Stimuli length	Mono vs. Poli5	−19.42	2.89	−25.07	−13.76	45.270	<0.001 *	0.602
		Mono vs. Poli4	−21.67	2.94	−27.42	−15.91	54.428	<0.001 *	0.671
		Mono vs. Tri	−20.67	2.63	−25.82	−15.51	61.675	<0.001 *	0.640
		Mono vs. Di	−12.29	2.00	−16.22	−8.37	37.698	<0.001 *	0.381

Note: 95% CI = 95% confidence interval; LL = lower limit; UL = upper limit; * = statistically significant value 5% (*p* ≤ 0.05); degrees of freedom = 1 for all analysis.

**Table 5:** Post hoc analyses of stimuli length according to type of stimuli and grade.

Grade	Comparison	Nonword						Word					
		Mean Difference	SE	95% CI		t	*p*	Mean Difference	SE	95% CI		t	*p*
				LL	UL					LL	UL		
1st Grade	Poli5 vs. Poli4	−1.62	5.54	−12.48	9.23	0.293	>0.999	2.25	6.84	−11.15	15.65	0.329	>0.999
	Poli5 vs. Tri	−8.77	5.87	−20.28	2.74	1.494	>0.999	1.25	6.60	−11.69	14.19	0.189	>0.999
	Poli5 vs. Di	−15.37	6.68	−28.47	−2.28	2.302	0.257	−7.12	7.20	−21.24	6.99	0.989	>0.999
	Poli5 vs. Mono	−19.17	6.72	−32.33	−6.00	2.854	0.064	−19.42	7.84	−34.77	−4.06	2.478	0.168
	Poli4 vs. Tri	−7.15	5.78	−18.47	4.17	1.238	>0.999	−1.00	6.32	−13.38	11.38	0.158	>0.999
	Poli4 vs. Di	−13.75	6.60	−26.68	−0.82	2.085	0.424	−9.37	6.94	−22.99	4.24	1.350	>0.999
	Poli4 vs. Mono	−17.54	6.63	−30.54	−4.54	2.645	0.110	−21.67	7.60	−36.56	−6.77	2.852	0.064
	Tri vs. Di	−6.60	6.88	−20.08	6.88	0.960	>0.999	−8.38	6.71	−21.53	4.78	1.248	>0.999
	Tri vs. Mono	−10.39	6.91	−23.94	3.16	1.503	>0.999	−20.67	7.39	−35.14	−6.19	2.798	0.074
	Di vs. Mono	−3.79	7.61	−18.71	11.13	0.498	>0.999	−12.29	7.93	−27.83	3.25	1.550	>0.999
2nd Grade	Poli5 vs. Poli4	−1.00	4.96	−10.72	8.72	0.202	>0.999	4.00	5.81	−7.40	15.40	0.688	>0.999
	Poli5 vs. Tri	−12.39	5.06	−22.30	−2.47	2.448	0.181	3.77	5.78	−7.55	15.10	0.653	>0.999
	Poli5 vs. Di	−31.50	5.59	−42.46	−20.54	5.635	<0.001 *	−6.00	5.55	−16.87	4.87	1.082	>0.999
	Poli5 vs. Mono	−31.42	5.35	−41.90	−20.93	5.874	<0.001 *	−20.83	5.52	−31.66	−10.01	3.773	0.004 *
	Poli4 vs. Tri	−11.39	4.57	−20.34	−2.43	2.493	0.162	−0.23	5.63	−11.27	10.81	0.040	>0.999
	Poli4 vs. Di	−30.50	5.15	−40.59	−20.41	5.923	<0.001 *	−10.00	5.39	−20.57	0.57	1.854	0.699
	Poli4 vs. Mono	−30.42	4.89	−39.99	−20.84	6.226	<0.001 *	−24.83	5.37	−35.36	−14.31	4.625	<0.001 *
	Tri vs. Di	−19.11	5.24	−29.39	−8.84	3.645	0.007 *	−9.77	5.35	−20.27	0.72	1.825	0.742
	Tri vs. Mono	−19.03	4.99	−28.80	−9.26	3.817	0.004 *	−24.61	5.33	−35.05	−14.16	4.616	<0.001 *
	Di vs. Mono	0.08	5.52	−10.74	10.91	0.015	>0.999	−14.83	5.08	−24.78	−4.88	2.922	0.053
3rd Grade	Poli5 vs. Poli4	−0.38	4.42	−9.04	8.29	0.085	>0.999	−0.63	3.41	−7.30	6.05	0.183	>0.999
	Poli5 vs. Tri	−9.80	4.29	−18.20	−1.39	2.285	0.267	−1.57	3.39	−8.21	5.07	0.463	>0.999
	Poli5 vs. Di	−27.63	4.32	−36.10	−19.15	6.389	<0.001 *	−2.75	3.31	−9.23	3.73	0.832	>0.999
	Poli5 vs. Mono	−21.92	4.43	−30.60	−13.23	4.944	<0.001 *	−9.75	3.36	−16.34	−3.16	2.899	0.056
	Poli4 vs. Tri	−9.42	3.34	−15.97	−2.87	2.818	0.070	−0.94	3.15	−7.12	5.23	0.299	>0.999
	Poli4 vs. Di	−27.25	3.39	−33.90	−20.60	8.037	<0.001 *	−2.13	3.06	−8.12	3.87	0.694	>0.999
	Poli4 vs. Mono	−21.54	3.53	−28.46	−14.63	6.105	<0.001 *	−9.12	3.12	−15.24	−3.01	2.923	0.053
	Tri vs. Di	−17.83	3.22	−24.13	−11.53	5.545	<0.001 *	−1.18	3.04	−7.14	4.77	0.389	>0.999
	Tri vs. Mono	−12.12	3.36	−18.71	−5.53	3.607	0.007 *	−8.18	3.10	−14.26	−2.10	2.638	0.112
	Di vs. Mono	5.71	3.41	−0.97	12.39	1.675	>0.999	−7.00	3.01	−12.90	−1.10	2.325	0.243
4th Grade	Poli5 vs. Poli4	4.62	3.66	−2.54	11.79	1.265	>0.999	3.63	2.44	−1.15	8.40	1.488	>0.999
	Poli5 vs. Tri	−2.32	3.56	−9.30	4.67	0.650	>0.999	1.23	2.05	−2.80	5.25	0.598	>0.999
	Poli5 vs. Di	−16.25	3.82	−23.75	−8.75	4.249	<0.001 *	3.25	1.90	−0.48	6.98	1.706	0.945
	Poli5 vs. Mono	−13.50	3.74	−20.82	−6.18	3.613	0.007 *	−1.50	1.92	−5.25	2.25	0.783	>0.999
	Poli4 vs. Tri	−6.94	3.03	−12.89	−1.00	2.290	0.265	−2.40	2.36	−7.03	2.24	1.014	>0.999
	Poli4 vs. Di	−20.88	3.33	−27.41	−14.34	6.262	<0.001 *	−0.38	2.24	−4.76	4.01	0.168	>0.999
	Poli4 vs. Mono	−18.13	3.23	−24.46	−11.79	5.608	<0.001 *	−5.12	2.25	−9.53	−0.72	2.282	0.269
	Tri vs. Di	−13.93	3.23	−20.27	−7.60	4.311	<0.001 *	2.02	1.81	−1.53	5.58	1.116	>0.999
	Tri vs. Mono	−11.18	3.13	−17.31	−5.05	3.576	0.008 *	−2.73	1.82	−6.30	0.85	1.495	>0.999
	Di vs. Mono	2.75	3.42	−3.95	9.45	0.804	>0.999	−4.75	1.65	−7.99	−1.51	2.871	0.061
5th Grade	Poli5 vs. Poli4	1.00	4.18	−7.20	9.20	0.239	>0.999	1.13	1.70	−2.22	4.47	0.660	>0.999
	Poli5 vs. Tri	−5.59	3.93	−13.29	2.11	1.423	>0.999	0.55	1.86	−3.09	4.18	0.294	>0.999
	Poli5 vs. Di	−17.25	3.96	−25.02	−9.48	4.352	<0.001 *	1.88	1.53	−1.12	4.87	1.227	>0.999
	Poli5 vs. Mono	−16.17	4.02	−24.05	−8.28	4.017	0.002 *	−0.67	1.76	−4.11	2.78	0.379	>0.999
	Poli4 vs. Tri	−6.59	3.09	−12.64	−0.54	2.135	0.379	−0.58	1.74	−4.00	2.84	0.332	>0.999
	Poli4 vs. Di	−18.25	3.13	−24.39	−12.11	5.826	<0.001 *	0.75	1.39	−1.97	3.47	0.540	>0.999
	Poli4 vs. Mono	−17.17	3.21	−23.46	−10.88	5.350	<0.001 *	−1.79	1.64	−5.00	1.42	1.094	>0.999
	Tri vs. Di	−11.66	2.78	−17.12	−6.20	4.188	0.001 *	1.33	1.57	−1.75	4.41	0.846	>0.999
	Tri vs. Mono	−10.58	2.87	−16.20	−4.95	3.686	0.006 *	−1.21	1.79	−4.73	2.30	0.676	>0.999
	Di vs. Mono	1.08	2.92	−4.64	6.80	0.371	>0.999	−2.54	1.45	−5.39	0.31	1.749	0.867

* = statistically significant value 5% (*p* ≤ 0.05).

## Data Availability

All data are available in excel arquives and also the videos that were recorded during the research conduction in the Research Laboratory where the study was conducted.

## Appendix A. List of Words

**Monosyllables**	**Disyllables**	**Trissylables**	**Polissylables**	**Polissilables-5+**
Boi	Noite	Trânsito	Tirânico	Característica
Fim	Caixa	Escola	Aquarela	Prejudicial
Luz	Chuva	Repolho	Enxurrada	Maravilhosa
Mar	Cedo	Brinquedo	Reciclagem	Experiência
Pé	Fala	Galinha	Exclamação	Insegurança
Zôo	Brinco	Zeloso	Exercício	Representação
	Depois	Canguru	Monarquia	Relaxamento
	Galho	Caracol	Nascimento	Reciprocidade
	Gente	Decisão	Obstáculo	
	Letra	Abençoar	Personagem	
	Barril	Guitarra	Satisfação	
	Vila	Exato	Criminoso	
	Feliz	Exceção	Vigilante	
	Texto	Expresso	Companheiro	
	Boxe	Orgulho	Cadeado	
	Peço	Frequência	Abóbora	
		Guerreiro		
		Carteira		
		Açúcar		
		Salada		
		Tóxico		
		Xícara		

## Appendix B. List of Non-Words

**Monosyllables**	**Disyllables**	**Trissylables**	**Polissylables**	**Polissilables-5+**
Doi	Neipe	Crânsupo	Purâmipe	Talactorústipa
Xim	Caufa	Ostolha	Apialolha	Trojubichual
Lhuz	Fiva	Recole	Onsirrega	Narajulesa
Nar	Chede	Drintodo	Refutlavom	Escoliônchia
Té	Felha	Baluna	Ostlanefão	Unfebirancha
Jôo	Drunco	Jelevo	Ejerfúchie	Retrosompafão
	Dequeis	Tambirá	Nemarpua	Relhassamonque
	Balhe	Paratel	Maschumompe	Rechuprecibade
	Genco	Defujão	Obspátulhe	
	Lopra	Agonfoar	Torfenavem	
	Garrul	Duparra	Sapuschafão	
	Vulha	Ovapo	Trunimeso	
	Cheluz	Ofechão	Visulhampo	
	Texque	Estrofo	Pentanoiro	
	Doxo	Erdulo	Tagoabe	
	Pofo	Fropenchia	Adéguela	
		Derroilo		
		Parcoira		
		Afútar		
		Chalega		
		Póxite		
		Fútara		
